# Supplementary material for: Content-rich biological network constructed by mining PubMed abstracts
Source: BMC Bioinformatics. 2004 Oct 8;5:147. doi: 10.1186/1471-2105-5-147 (PMC528731; doi:10.1186/1471-2105-5-147)
Supplement: Additional File 2 — The original results of the above study (non-essential files are deleted to keep the file size under the limit set by BMC bioinformatics). [file 1471-2105-5-147-S2.bz2 › chilibotAdditionalFile2/dip05/458ID10488147E10476/html/CCR3_CCL26.html]

 


 **CCR3** and **CCL26** 
  
Found 20 abstracts in PubMed, retrieved 05.  
 

 What does Google say? 
 PDF only 
| .edu only 

---

**Interactive relationship** (e.g. stimulation, inhibition, etc)

**Neutral relationship**- Like eotaxin and eotaxin 2, eotaxin 3  [ **CCL26** ]  mediates its activity through  **CCR3** .  Ref: 12689946 Blood< MedlineTA>Blood,
- The Eotaxin gene family Eotaxin1, Eotaxin2 and Eotaxin3  [ **CCL26** ]  recruits and activates  **CCR3**  bearing cells such as eosinophils, mast cells and Th2 lymphocytes that play a major role in allergic disorders.  Ref: 12761043 Hum Mol Genet< MedlineTA>Hum Mol Genet,

**Non-interactive relationship** (e.g. studied together, co-existance, homology, etc.)

- In contrast, the  **CCR3**  agonists CCL11, CCL24 and  **CCL26**  are involved in the recruitment of cells such as eosinophils and basophils during Th2 responses.  Ref: 12884299 Eur J Immunol, 2003
